# Reversible electrochemical oxidation of sulfur in ionic liquid for high-voltage Al−S batteries

**DOI:** 10.1038/s41467-021-26056-7

**Published:** 2021-09-29

**Authors:** Huan Li, Rongwei Meng, Yong Guo, Biao Chen, Yan Jiao, Chao Ye, Yu Long, Anton Tadich, Quan-Hong Yang, Mietek Jaroniec, Shi-Zhang Qiao

**Affiliations:** 1 School of Chemical Engineering and Advanced Materials, The University of Adelaide, Adelaide, SA 5005 Australia; 2 Nanoyang Group, State Key Laboratory of Chemical Engineering, School of Chemical Engineering and Technology, Tianjin University, 300072 Tianjin, China; 3 School of Materials Science and Engineering and Tianjin Key Laboratory of Composite and Functional Materials, Tianjin University, 300350 Tianjin, China; 4 Australian Synchrotron (ANSTO), 800 Blackburn Road, Clayton, VIC 3168 Australia; 5 Department of Chemistry and Biochemistry & Advanced Materials and Liquid Crystal Institute, Kent State University, Kent, OH 44242 USA

**Keywords:** Batteries, Batteries

## Abstract

Sulfur is an important electrode material in metal−sulfur batteries. It is usually coupled with metal anodes and undergoes electrochemical reduction to form metal sulfides. Herein, we demonstrate, for the first time, the reversible sulfur oxidation process in AlCl_3_/carbamide ionic liquid, where sulfur is electrochemically oxidized by AlCl_4_^−^ to form AlSCl_7_. The sulfur oxidation is: 1) highly reversible with an efficiency of ~94%; and 2) workable within a wide range of high potentials. As a result, the Al−S battery based on sulfur oxidation can be cycled steadily around ~1.8 V, which is the highest operation voltage in Al−S batteries. The study of sulfur oxidation process benefits the understanding of sulfur chemistry and provides a valuable inspiration for the design of other high-voltage metal−sulfur batteries, not limited to Al−S configurations.

## Abstract (web summary)

The sulfur cathode in metal-sulfur batteries normally undergoes electrochemical reduction to form metal sulfides. Here, the authors demonstrate the electrochemical oxidation of sulfur in ionic liquid for high-voltage aluminium-sulfur batteries.

## Introduction

Sulfur is a promising electrode material in metal–sulfur batteries due to its earth abundance and high theoretical capacity^1–6^. Sulfur is normally coupled with metal anodes and is electrochemically reduced with metal cations to form metal sulfides^7,8^. Despite high specific capacities based on sulfur reduction, the reverse oxidation of these sulfides back to sulfur needs to overcome a high energy barrier^9,10^, leading to a large overpotential and poor reversibility. Additionally, the reduction of sulfur occurs at low electrochemical potential (~−1.0 V vs. standard hydrogen electrode, Fig. S1), and results in low operation voltage of metal–sulfur batteries^5,6^. For example, Al–S batteries based on sulfur reduction usually demonstrate ultralow cell voltage of about ~0.5 V^11,12^. Therefore, the limited reversibility and low electrochemical potentials are the main obstacles for the practical use of sulfur electrodes.

Many efforts have been devoted toward improving the reversibility of metal–sulfur batteries by proper designs of sulfur host and electrolyte engineering^5,6,13–15^. However, these prior attempts failed to basically address the low-voltage concerns of metal–sulfur batteries because the cell voltage is determined by the redox pathway of sulfur but these efforts did not alter the sulfur reduction path. Therefore, the batteries based on the sulfur reduction remain far from satisfactory for the high-voltage applications^5–8,16–18^. It is highly important to examine new redox pathways of sulfur to achieve viable applications of metal–sulfur batteries. In this regard, sulfur oxidation is a worthy path because it can compensate the intrinsic low-voltage shortcoming of sulfur reduction.

Considering the multivalent nature of sulfur element (−2, 0, +2, +4, +6), sulfur can be oxidized into high-valence sulfur compounds^19,20^. Unfortunately, the oxidation process of sulfur has been rarely studied. Due to the inert nature of sulfur, a high voltage needs to be applied to drive its electrochemical oxidation. This is normally accompanied by electrolyte decomposition, leading to a poor reversibility^21–23^. Meanwhile, the electrochemical oxidation of sulfur undergoes an electron-loss process involved with anions. The common anions in metal–sulfur batteries such as bis (trifluoromethyl) sulfonate, hexafluorophosphate are weak oxidants, which are not able to oxidize sulfur into high-valence sulfur compounds^24,25^. Therefore, anions with strong oxidizing power in an electrochemically stable electrolyte are necessary to oxidize sulfur in a highly reversible manner, but this concept has not been explored yet.

In this work, we demonstrate, for the first time, the reversible sulfur oxidation in AlCl_3_/carbamide ionic liquid. The AlCl_4_^−^ anions can oxidize sulfur to form aluminium sulfide chloride (AlSCl_7_), which can be reversibly reduced back to sulfur with a high efficiency of ~94%. This oxidation–reduction process is workable within a wide range of high electrochemical potentials. Benefiting from the high reversibility and high electrochemical potential, the Al–S battery can run steadily over 200 cycles around ~1.8 V, which is the highest operation voltage in Al–S batteries reported so far. By sharp contrast, the previously studied Al–S battery based on sulfur reduction can only run tens of cycles with a much lower operation voltage of ~0.5 V. This work sheds new light on the understanding of sulfur chemistry and presents sulfur oxidation as a new pathway to achieve the high-voltage applications of metal–sulfur batteries.

## Results

### Evolution of AlSCl_7_ during electrochemical sulfur oxidation

**Fig. 1:**
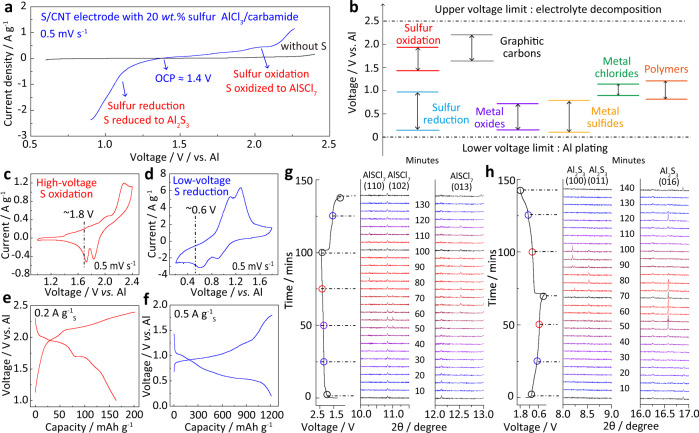
The electrochemical oxidation and reduction of sulfur in ionic liquid. **a** LSV curves of S/CNT composite cathode in AlCl_3_/carbamide ionic liquid at 0.5 mV s^−1^ with Al referenced electrode; the sulfur content in S/CNT is 20 wt.%; **b** the voltage comparison of sulfur oxidation and sulfur reduction with previously reported materials; CV curves of **c** sulfur oxidation and **d** sulfur reduction at 0.5 mV s^−1^; galvanostatic charge–discharge curves of the S/CNT cathode based on **e** sulfur oxidation at 0.2 A g^−1^ and **f** sulfur reduction at 0.5 A g^−1^; the time-dependent in situ synchrotron-based XRD patterns for **g** sulfur oxidation and **h** sulfur reduction processes and the corresponding charge–discharge curves. The current densities for sulfur oxidation and sulfur reduction are 0.2 and 0.5 A g^−1^, respectively.

AlSCl_7_ is an ionic crystal with SCl_3_^+^ cations and AlCl_4_^−^ anions^26^. AlCl_3_/carbamide ionic liquid contains AlCl_4_^−^, Al_2_Cl_7_^−^ anions, and [AlCl_2_(carbamide)_*n*_]^+^ cations^27,28^. Al anode demonstrates high reversibility and stable electrochemical potential in AlCl_4_^−^ and Al_2_Cl_7_^−^ containing electrolyte, and therefore Al anode is used as the reference electrode in this work^29–32^. Figure 1a shows the linear scanning voltammetry (LSV) curve for sulfur/carbon nanotube (S/CNT) composite cathode with 10 wt.% polyvinylidene difluoride (PVDF) binder in AlCl_3_/carbamide electrolyte (Fig. S2). It should be noted that sulfur is not stable under high oxidation voltage in the commonly used AlCl_3_/1-ethyl-3-methylimidazolium chloride electrolyte (Fig. S3). The sulfur content in S/CNT is 20 wt.% and the molar ratio of AlCl_3_ to carbamide is 1.3:1 (details in the “Methods” section). The observed current densities in Fig. 1a are above ~2.0 V and below ~1.0 V, corresponding to the electrochemical oxidation and reduction of sulfur, respectively. Figure 1b summarizes the electrochemical potentials of different materials, and the potential of sulfur oxidation in this work is much higher than most of the previously reported materials (Table S1)^11,12^. Cyclic voltammetry (CV) curves are compared to demonstrate the overall process of sulfur oxidation and reduction. The electrochemical oxidation from sulfur to AlSCl_7_ starts from ~2.0 V, and the reverse reduction from AlSCl_7_ to sulfur occurs at ~1.8 V (Fig. 1c). For the sulfur reduction (Fig. 1d), sulfur starts to be reduced to sulfides below ~1.0 V. The electrochemical potential of sulfur oxidation is much higher than that of reduction. The potential difference is clearly seen in the galvanostatic charge–discharge curves. The sulfur oxidation presents obviously high discharge voltage plateau of ~1.8 V (Fig. 1e). However, the reduction of sulfur only shows a much lower operation voltage of ~0.5 V (Fig. 1f). To detect the phase evolution during the oxidation and reduction process, we carried out the in situ synchrotron-based X-ray diffraction (XRD) upon charge and discharge (Fig. 1g, h). The elemental sulfur, Al_2_S_3_, and AlSCl_7_ are orthorhombic, hexagonal, and monoclinic crystals with space groups of Fddd, P6_1_, and Pc, respectively (Fig. S4). During sulfur oxidation process, the orthorhombic sulfur transforms to the monoclinic AlSCl_7_, while during reduction process, sulfur is reduced to hexagonal Al_2_S_3_. Specifically, for sulfur oxidation process (Fig. 1g), the peaks assigned to the (102), (013), and (110) facets of AlSCl_7_ sequentially appear when the Al–S battery is charged to 2.4 V. For the reverse process from AlSCl_7_ to sulfur, those peaks assigned to AlSCl_7_ gradually disappear, and only the characteristic peaks of sulfur remain. It should be noted that the diffraction peak at 10.8° is assigned to the characteristic peak of sulfur and it remains during charge–discharge due to the incomplete electrochemical oxidation of sulfur. The above evidence confirms the efficient electrochemical oxidation of sulfur to AlSCl_7_, and the reversibility from AlSCl_7_ back to sulfur. During sulfur reduction process (Fig. 1h), the (011) and (016) diffraction peaks of Al_2_S_3_ appear at 8.5^o^ and 16.6^o^, respectively. For the reverse charge process, these peaks gradually disappear due to the conversion of Al_2_S_3_ to sulfur. However, the (100) and (016) characteristic peaks of Al_2_S_3_ can be also found during the charge process, which is due to the difficulty of reversible decomposition of Al_2_S_3_ to sulfur. Both AlSCl_7_ and Al_2_S_3_ are also verified by the XRD plots at different cut-off voltages (Fig. S5). The above evidence confirms sulfur oxidation and reduction chemistry, which is based on the AlSCl_7_ and Al_2_S_3_ products, respectively.

**Fig. 2:**
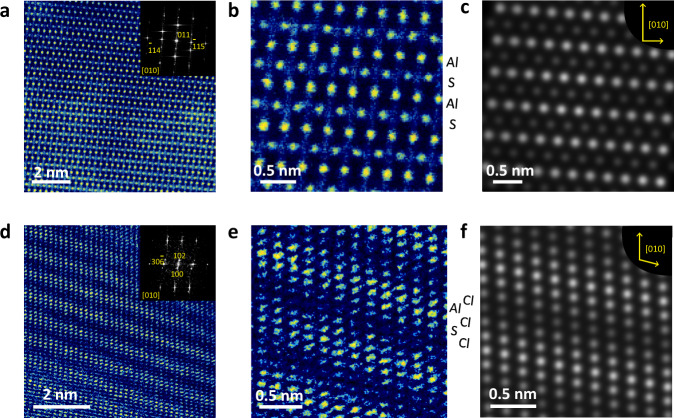
Microscopic images of reduced and oxidized products of sulfur. **a**, **b** STEM image of Al_2_S_3_, and the inset in **a** shows the FFT patterns; **c** the simulated microscopic image of Al_2_S_3_ observed from [010] direction; **d**, **e** high-resolution TEM images of AlSCl_7_, and the inset in **d** shows the FFT patterns; (**f**) the simulated microscopic image of AlSCl_7_ observed from [010] direction.

A direct view of these products is shown on the scanning transmission electron microscopic (STEM) images after charging the sulfur cathode at 2.4 V and discharging at 0.2 V. S_8_ octamer is visible on the TEM image of pristine sulfur (Fig. S6). After electrochemical S reduction at 0.2 V (Fig. 2a), a crystallized structure is seen with ($\bar{1}14$), ($\bar{1}15$), and (011) planes of Al_2_S_3_ in the Fast Fourier Transform (FFT) patterns. The high-resolution image presents an orthogonal arrangement of atoms (Fig. 2b), corresponding to the simulated Al_2_S_3_ images from [010] observation (Figs. 2c and S4). By comparison, the oxidized product of sulfur at 2.4 V shows a periodic layered structure with (100), (102), and ($30\bar{6}$) planes in the FFT patterns. The observation of (102) plane corresponds well with the in situ XRD patterns. The ordered atom distribution can be clearly identified in Fig. 2e. Al, S, and Cl atoms are orderly arranged, coinciding well with simulated AlSCl_7_ images from [010] observation (Figs. 2f and S7). The Al, S, and Cl atoms can be also identified by the elemental mappings (Fig. S8). These results well characterize the phase evolution for the oxidation and reduction process of sulfur, where AlSCl_7_ and Al_2_S_3_ are the main products.

### Theoretical computations of reaction pathways

**Fig. 3:**
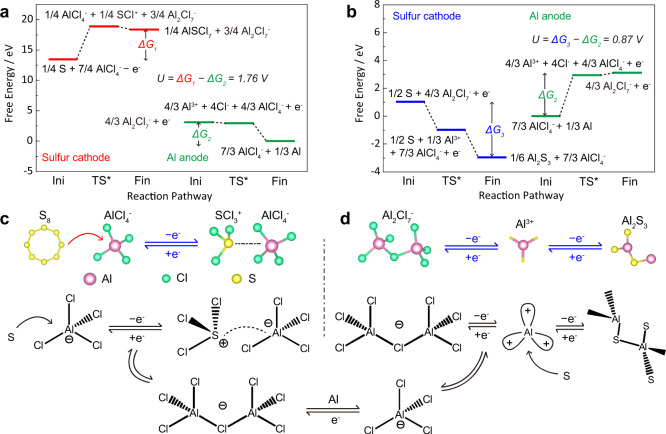
Theoretical computations of reaction pathways for sulfur oxidation and sulfur reduction. The reaction pathway of sulfur cathode and Al anode for **a** sulfur oxidation and **b** sulfur reduction; schematics for **c** sulfur oxidation and **d** sulfur reduction processes with AlCl_4_^−^ and Al_2_Cl_7_^−^ anions, respectively.

To give an insight into the pathways of sulfur oxidation and reduction, we simulate the interactions between sulfur and AlCl_4_^−^ and Al_2_Cl_7_^−^ cations based on density functional theory (DFT). The DFT-based energy, zero-point energy, entropy, and Gibbs free energy for all the intermediates are listed in Table S2, and their optimized structures are shown in Fig. S9. The details for determination of the Gibbs free energy are specified in the “Theoretical computations” part. For the sulfur oxidation process (Fig. 3a), AlCl_4_^−^ oxidizes S into AlSCl_7_ solid accompanied by the loss of electrons (Eq. 1). Meanwhile, Al_2_Cl_7_^−^ is reduced into AlCl_4_^−^ with Al plated on Al anode. The change in the Gibbs free energy (ΔG) can be calculated by using the electron-transfer numbers (*n*) and the difference in the electrochemical potential (Δ*U*). With single-electron transfer, the difference in ΔG between cathode and anode is the operation voltage^33^. As expected, the sulfur oxidation demonstrates a high voltage of ~1.76 V, which is consistent with the experimental observations of ~1.8 V (Fig. 1c). For the sulfur reduction process, sulfur is reduced by Al_2_Cl_7_^−^ cations to form Al_2_S_3_ (Eq. 2), and meanwhile, AlCl_4_^−^ etches Al anode to form Al_2_Cl_7_^−^. The voltage based on sulfur reduction is only ~0.87 V (Fig. 3b), much lower than that of sulfur oxidation. Additionally, the reverse reduction from AlSCl_7_ to S only needs to overcome an energy barrier of 0.52 eV as calculated by the uphill of red lines in Fig. 3a. However, the energy barrier from Al_2_S_3_ to S is as high as 3.98 eV (blues lines in Fig. 3b). This comparison suggests the ease of reverse conversion from AlSCl_7_ to S and therefore better reversibility of the sulfur oxidation–reduction process. Figure 3c, d schematically compares the sulfur oxidation and reduction process. The AlCl_4_^−^ and Al_2_Cl_7_^−^ anions serve, respectively, as the oxidizing and reducing agents reacting with sulfur, and Al anode is used as referenced electrode to pair with these redox reactions for charge balance. We have summarized the pathways of sulfur oxidation and reduction as follows:

Sulfur oxidation:

1 $$\,\frac{1}{4}{{\mbox{S}}}+\frac{7}{4}{{\mbox{AlC}}}{{{\mbox{l}}}}_{4}^{-}-{{{\mbox{e}}}}^{-}\mathop{\longleftrightarrow }\limits^{{{\mbox{oxidation}}}}\frac{1}{4}{{\mbox{AlSC}}}{{{\mbox{l}}}}_{7}+\frac{3}{4}{{\mbox{A}}}{{{\mbox{l}}}}_{2}{{\mbox{C}}}{{{\mbox{l}}}}_{7}^{-}\,$$

Sulfur reduction:

2 $$\,\frac{1}{2}{{\mbox{S}}}+\frac{4}{3}{{\mbox{A}}}{{{\mbox{l}}}}_{2}{{\mbox{C}}}{{{\mbox{l}}}}_{7}^{-}\mathop{\longleftrightarrow }\limits^{{{\mbox{reduction}}}}\frac{1}{6}{{\mbox{A}}}{{{\mbox{l}}}}_{2}{{{\mbox{S}}}}_{3}+\frac{7}{3}{{\mbox{AlC}}}{{{\mbox{l}}}}_{4}^{-}+{{{\mbox{e}}}}^{-}$$

### Track of reaction intermediates via spectroscopic analysis

**Fig. 4:**
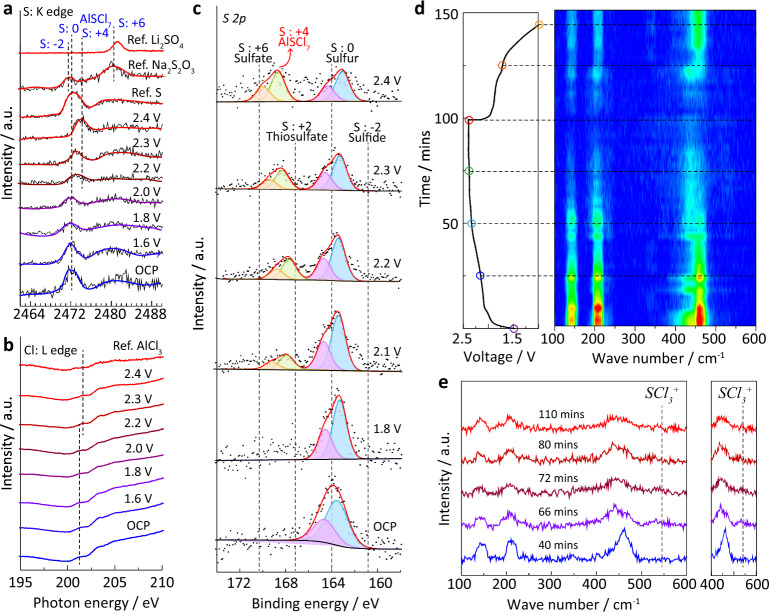
Identification of oxidized intermediates of sulfur via spectroscopic analysis. NEXAFS spectra of S/CNT cathode with different voltages recorded at the **a** S *K*-edge and **b** Cl *L*-edge; **c** the S 2p XPS spectra of S/CNT cathode at different oxidation voltages; the Al–S batteries were charged from open-circuit potential (OCP) to 2.4 V at 0.2 A g^−1^, and then the S/CNT cathodes at different cut-off voltages were extracted from the disassembled Al–S batteries for NEXAFS and XPS investigations; **d** the charge–discharge curves of Al–S batteries at 0.2 A g^−1^ and their real-time Raman contour map for S/CNT cathode; **e** Raman spectra at different times extracted from the contour pattern in **d**.

We combined synchrotron-based near-edge X-ray absorption fine structure (NEXAFS) spectra, X-ray photoelectron spectra (XPS), and in situ Raman spectra to identify the reaction intermediates during the sulfur oxidation process. As shown in the S *K*-edge NEXAFS spectra (Fig. 4a), the characteristic peak of sulfur located at ~2472 eV presents an obvious positive shift with higher voltage from open circuit potential (OCP) to 2.4 V. The shifted peak position of oxidation products from 2.2 to 2.4 V is nicely located between the peaks of 0-valence and +6-valence sulfur as compared to the reference samples of elemental sulfur, N_2_S_2_O_3_, and Li_2_SO_4_. This suggests the efficient oxidation of sulfur to higher valence at high voltages^34^. The positive shift of Cl characteristic peak is also identified from the Cl *L*-edge spectra at ~201.3 eV (Fig. 4b). This is attributed to the formation of S–Cl bonds in AlSCl_7_. The electronegativity of S is stronger than Al, and therefore the photon energy of Cl atoms among Cl–S bonds is higher than those among Cl–Al bonds^35^. By contrast, there is no peak shift for Al characteristic peak because the chemical state of Al remains unchanged during the sulfur oxidation process (Fig. S10). To quantify the sulfur valence during its oxidation, we carried out the XPS measurement at different oxidation voltages (Fig. 4c). According to the S 2p XPS spectra, peaks located at ~169.4 and ~168.3 eV gradually appear between the +2 thiosulfate and +6 sulfate^36^. These doublets are assigned to the +4 sulfur, confirming the stable presence of AlSCl_7_ oxidized products. To detect the oxidation intermediates, we further carried out in situ Raman spectra measurement (Fig. S11)^37^. As shown in Fig. 4d, Raman peaks located at ~145, ~210, and ~462 cm^−1^ are assigned to sulfur (Fig. S12)^38^. During the charging process, the intensity of these peaks gradually weakens, indicating the conversion from sulfur to AlSCl_7_. For the reverse process, these Raman peaks of sulfur reappear, which is a strong evidence of the high reversibility of sulfur oxidation. Notably, a new peak located at 530 cm^−1^ emerges (Fig. 4e), which is attributed to the vibration of SCl_3_^+^ cations^39^. These cations are soluble in the electrolyte, which may trap into separator and shuttle to Al anode. Therefore, the sulfur valence and reversibility are well characterized by spectroscopic analysis. AlSCl_7_ tends to decompose to AlCl_4_^−^ and SCl_3_^+^ intermediates, leading to the loss of sulfur during electrochemical cycling.

### High-voltage Al–S batteries based on sulfur oxidation

**Fig. 5:**
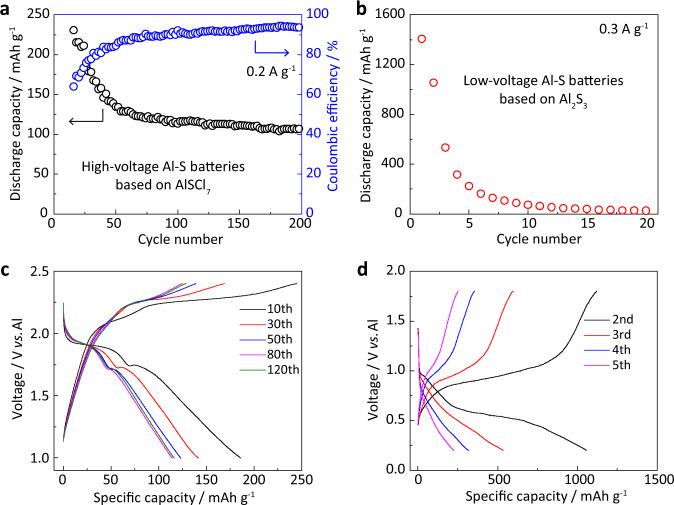
Sulfur oxidation in Al–S batteries. The cycling performance of Al–S batteries based on **a** sulfur oxidation, AlSCl_7_ products and **b** sulfur reduction, Al_2_S_3_ products; the charge–discharge curves at different cycles of Al–S batteries based on **c** sulfur oxidation and **d** sulfur reduction.

To demonstrate the possible application of sulfur oxidation, we assembled Al–S batteries based on the oxidation and reduction process and compared their operation voltages and cyclic stability. Al–S batteries were assembled with S/CNT cathode, Al referenced anode, AlCl_3_/carbamide ionic liquid, and a glass fiber separator using a 2032-coin cell type (more details in the “Electrochemical tests” section). The specific surface area of the cathode with 20 wt.% S in S/CNT composite is 154 m^2^ g^−1^ (Fig. S13). As shown in Fig. 5a, Al–S batteries based on the sulfur oxidation (AlSCl_7_ product) run steadily over 200 cycles with a highest specific capacity of 225 mAh g^−1^ (Fig. S14). Reversible redox reactions are defined as a pair of oxidation–reduction reactions with high reversibility. Coulombic efficiency (CE%) is a good parameter to describe the reversibility of electrochemical reactions on the electrodes in batteries. In this work, CE% is defined as the percentage ratio of the specific discharge capacity to the charge capacity. For Al–S batteries based on the sulfur oxidation, the CE% stabilizes as high as ~94% upon cycling. This is ascribed to the efficient electrochemical oxidation of sulfur to AlSCl_7_ and then highly reversible reduction from AlSCl_7_ back to sulfur. However, it should be noted that the Al–S battery based on the sulfur oxidation also exhibits capacity decay upon long cycles. This is attributed to the gradual dissolution of SCl_3_^+^ into the electrolyte (as evidenced by the in situ Raman spectra), leading to the loss of active sulfur (Figs. S15 and S16). Future work needs to be carried out to restrain the dissolution of SCl_3_^+^ for more stable cycling performance. For the previously studied batteries based on sulfur reduction with Al_2_S_3_ product, the sulfur cathode showed an ultrahigh specific capacity over 1000 mAh g^−1^ at the first cycle. However, it dramatically declined after tens of cycles due to the irreversibility (Fig. 5b)^40–42^. Figure 5c, d shows a comparison of the charge–discharge curves at different cycles. The Al–S battery based on the sulfur oxidation exhibits a high voltage of ~1.8 V, and the voltage plateau remains stable during cycling. However, the Al–S batteries based on the sulfur reduction feature a much lower voltage of ~0.5 V with severe voltage decline and capacity decay. Additionally, the Al–S battery based on the sulfur oxidation demonstrates high-rate performance. The sulfur cathode still has a high specific capacity of 120 and 95 mAh g^−1^ at 0.5 and 1 A g^−1^, respectively (Fig. S17). The Al–S battery maintains stable cycling at high current densities (Figs. S18 and S19). However, it is noteworthy that the specific capacity of sulfur decreases with higher sulfur contents and areal mass loadings (Figs. S20 and S21). Therefore, more attention should be paid in future on the design of sulfur host to improve the sulfur utilization with sulfur oxidation process.

Despite the above advantages of Al–S batteries, we should also evaluate their pros and cons. The maximized energy density based on the active sulfur is estimated at ~405 Wh kg^−1^ considering a specific capacity of 225 mAh g^−1^ with an average voltage plateau of ~1.8 V at the beginning cycles. However, it should be noted that this value decreases upon battery cycling, and the energy density will be also compromised while considering the practical devices, including Al anode, electrolyte, separator, cell case, etc. Further work still needs to be carried out to optimize the energy density by improving the sulfur utilization and the areal mass loading of sulfur while decreasing the dosage of non-active parts. In addition, another advantage of Al–S battery is the low cost of electrode materials such as sulfur, Al, and the AlCl_3_/carbamide electrolyte (Fig. S22 and Table S3). However, it should be also noted that the carbon nanotubes used in this work raise concerns about the cost-effectiveness. In the future works, it would be highly desirable to explore low-cost carbon or noncarbon hosts with lower cost and higher sulfur utilization.

## Discussion

We have demonstrated that sulfur can be electrochemically oxidized in ionic liquid with high reversibility. The reaction pathways, AlSCl_7_ oxidized products, and SCl_3_^+^ intermediates are well confirmed by means of in situ synchrotron-based analysis, high-resolution microscopic images, spectroscopic analysis, and theoretical computations. The electrochemical oxidation from sulfur to AlSCl_7_ is highly reversible with a stable CE% of ~94%, and the oxidation process is workable within a wide range of electrochemical potentials. As a result, the Al–S battery based on sulfur oxidation process can run steadily over 200 cycles around ~1.8 V, which is the highest operation voltage for Al–S batteries. It is expected that the sulfur oxidation process can be coupled with other metal anodes for various metal–sulfur batteries, not limited to Al–S batteries. This work sheds new light on sulfur chemistry and shows a great advantage of the sulfur oxidation pathway for the design of viable high-voltage metal–sulfur batteries.

## Methods

### Preparation of S/CNT cathode and AlCl_3_/carbamide ionic liquid

The S/CNT material was prepared by mixing sublimed S with CNT under 155 °C for 12 h. Different sulfur contents in S/CNT can be achieved by adjusting the relative mass ratio of S and CNT. In all, 20, 40, and 80 wt.% of sulfur were used in this work. The S/CNT cathode was prepared by mixing S/CNT material with PVDF binder with a mass ratio of 90:10. The AlCl_3_/carbamide ionic liquid was synthesized by mixing AlCl_3_ and carbamide with a molar ratio of 1.3:1. Specifically, AlCl_3_ was gradually added into carbamide with continuous stirring in an Ar-filled glove box at the room temperature. During this process, these two solids melt with each other into liquid and finally form ionic liquid containing AlCl_4_^−^, Al_2_Cl_7_^−^, and [AlCl_2_(carbamide)_*n*_]^+^.

### Characterization of materials

The morphology and structure of the samples were characterized by scanning electron microscopy (Hitachi S4800, Japan). High-resolution TEM and STEM images were obtained by JEM-ARM200F TEM. XPS spectra were measured with the Thermo Fisher Scientific ESCALAB Xi+, Al Kα radiation. NEXAFS of S *K*-edge and Cl *L*-edge were performed on the soft X-ray spectroscopy beamline at Australian synchrotron (Clayton) AS, part of ANSTO. In situ synchrotron XRD data were collected on the powder diffraction beamline at the Australian Synchrotron with a wavelength of 0.6868 and 0.7290 Å. Data were collected continuously in 30 s acquisitions with coin cells. For sulfur oxidation, first the cells were charged at 0.2 A g^−1^ to 2.4 V and then discharged to 1.0 V. For sulfur reduction, first the cells were discharged to 0.5 V and then charged to 1.8 V at 0.5 A g^−1^. The cell cases on both the negative and positive sides together with the Al foil anode were punched with *d* = 0.2 cm holes, and polyimide films were used to seal the holes but allowed the X-ray transmission. In situ Raman spectra were collected with Labram HR Evolution (Horiba scientific).

### Electrochemical tests

For the assembly of Al–S batteries, the as-prepared S/CNT electrodes with different sulfur contents were coupled with an Al foil reference anode (100 μm thickness). These two electrodes were sandwiched by a glass fiber separator (GF/A) with AlCl_3_/carbamide ionic liquid (~140 μL). These components were placed into a 2032-coin cell configuration for further electrochemical tests. The LSV curves were scanned from OCP (≈1.4 V) at 0.5 mV s^−1^. CV was carried out from 1.0 to 2.4 V for sulfur oxidation and from 0.2 V to 1.8 V for sulfur reduction. Data of LSV and CV curves were collected on an IVIUM electrochemical workstation. Galvanostatic charge–discharge cycles were performed at different current densities using a Neware battery tester.

### Theoretical computations

Computations for this work were carried out using DFT as implemented in VASP code. Electronic exchange–correlation energy was modeled using the Perdew–Burke–Ernzerhof function within a generalized gradient approximation. The projector-augmented wave method was used to describe the ionic cores. For the plane-wave expansion, a 450 eV kinetic energy cut-off was used following testing a series of different cut-off energies. Convergence criterion for the electronic structure iteration was set to 10^−4^ eV and that for geometry optimization was 0.01 eV Å^−1^ on force. A Gaussian smearing of 0.1 eV was applied during geometry optimization and for total energy computations.

The Gibbs free energy was calculated based on the DFT-based energy (*E*), zero-point energy (ZPE), and the entropy (TS) by using the following expression:

3 $$G\,=\,E+{{{{{\rm{ZPE}}}}}}-{{{{{\rm{TS}}}}}}$$

The change in the Gibbs free energy (Δ*G*) can be calculated by using the electron-transfer numbers (*n*) and the difference in the electrochemical potential (Δ*U*).

4 $$\Delta G=-{{{{{\rm{ne}}}}}}\Delta U$$

The difference in Δ*G* between cathode and anode is the cell operation voltage with single-electron transfer (*n* = 1).

## Supplementary information

Supplementary Information

## Data Availability

The data that support the findings of this study are available from the corresponding author upon reasonable request.
