# Supplementary material for: Reversible electrochemical oxidation of sulfur in ionic liquid for high-voltage Al−S batteries
Source: Nat Commun. 2021 Sep 29;12:5714. doi: 10.1038/s41467-021-26056-7 (PMC8481422; doi:10.1038/s41467-021-26056-7)
Supplement: Supplementary file 1 — Supplementary Information [file 41467_2021_26056_MOESM1_ESM.pdf]

Supplementary Information

Reversible electrochemical oxidation of sulfur in ionic liquid for high-voltage Al−S batteries

Huan Li[1†], Rongwei Meng[2†], Yong Guo[2], Biao Chen[3], Yan Jiao[1], Chao Ye[1], Yu Long[2], Anton Tadich,[4] Quan-Hong Yang[2], Mietek Jaroniec[5] and Shi-Zhang Qiao[*1]

[1]School of Chemical Engineering and Advanced Materials, The University of Adelaide, Adelaide, SA 5005, Australia;

[2]Nanoyang Group, State Key Laboratory of Chemical Engineering, School of Chemical Engineering and Technology, Tianjin University, Tianjin 300072, China;

[3]School of Materials Science and Engineering and Tianjin Key Laboratory of Composite and Functional Materials, Tianjin University, Tianjin 300350, China.

[4]Australian Synchrotron (ANSTO), 800 Blackburn Rd, Clayton, VIC 3168, Australia

[5]Department of Chemistry and Biochemistry & Advanced Materials and Liquid Crystal Institute, Kent State University, Kent, OH 44242, USA.

[†]These authors contribute equally to this work.

* Corresponding author e-mail: s.qiao@adelaide.edu.au

Supplementary Results

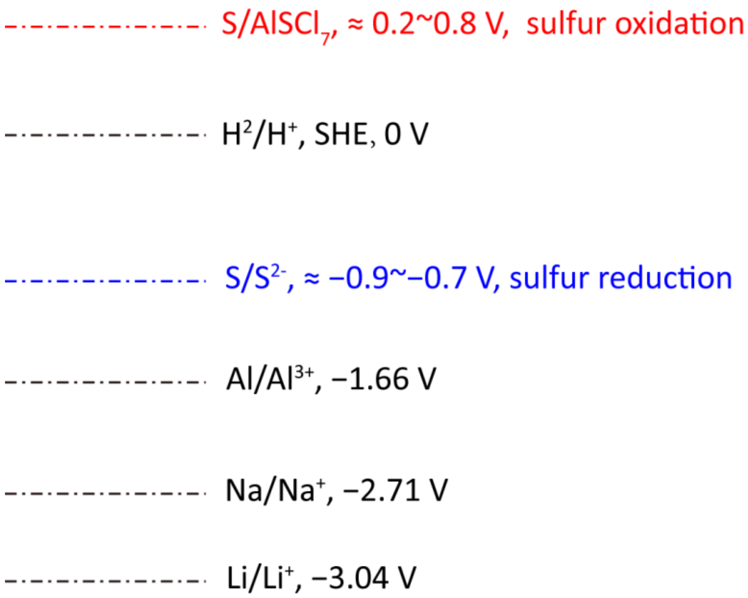

Supplementary Figure S1 The estimated equilibrium electrochemical potentials of sulfur reduction and sulfur oxidation.

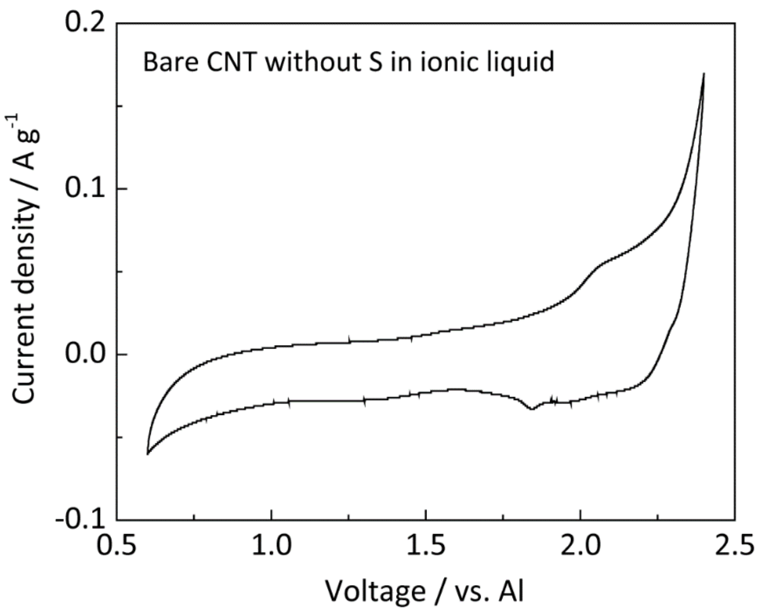

Supplementary Figure S2 CV curves of bare CNT in $AlCl_3$/urea with a scan rate of 0.5 mV s^{-1}. The CV data show much lower current density (y axis), suggesting the low electrochemical activity of CNT in ionic liquid.

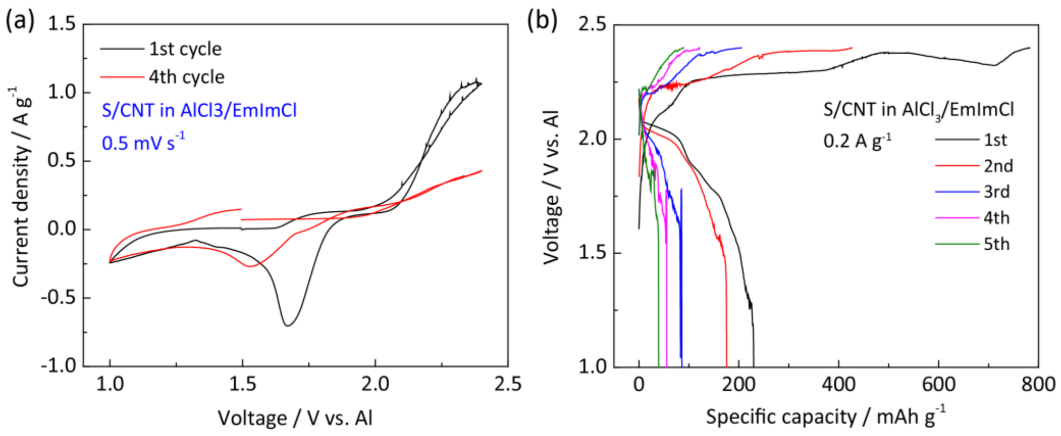

Supplementary Figure S3 The electrochemical oxidation of sulfur in $AlCl_3$/EmimCl electrolyte. The molar ratio of $AlCl_3$ to EmimCl is 1.3:1. (a) CV curves of S/CNT from 1.0 V to 2.4 V at a scan rate of 0.5 mV s^{-1}. The severely decreased peak currents can be seen from the 1st cycle and 4th cycle, indicating the poor reversibility of sulfur in this electrolyte. (b) Charge-discharge curves of S/CNT from 1.0 V to 2.4 V at 0.2 A g^{-1}. The discharge capacity dramatically decreases from 230 mAh g^{-1} to 41 mAh g^{-1} from 1st to 5th cycles, confirming the instability of sulfur in this electrolyte during electrochemical oxidation.

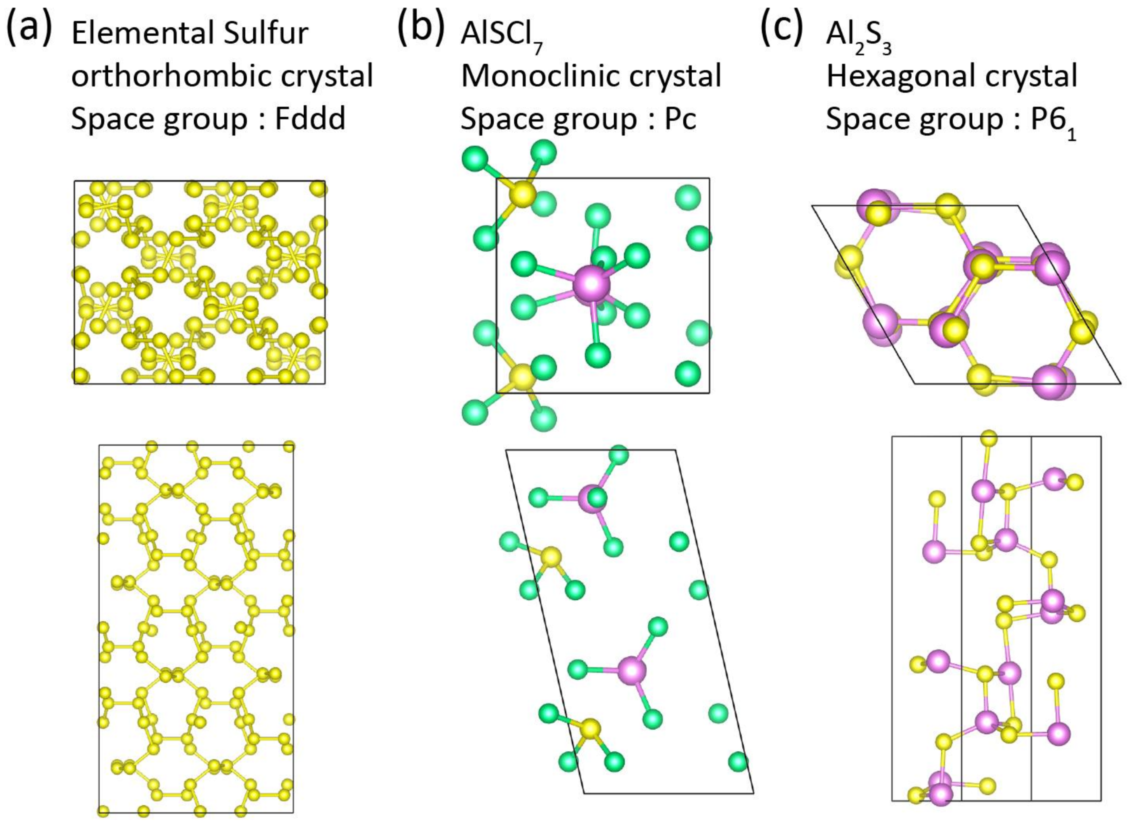

Supplementary Figure S4 The models, crystal structures and space groups of elemental sulfur, $AlSCl_7$ and Al_2S_3. The purple, yellow and green atoms denote Al, S and Cl, respectively.

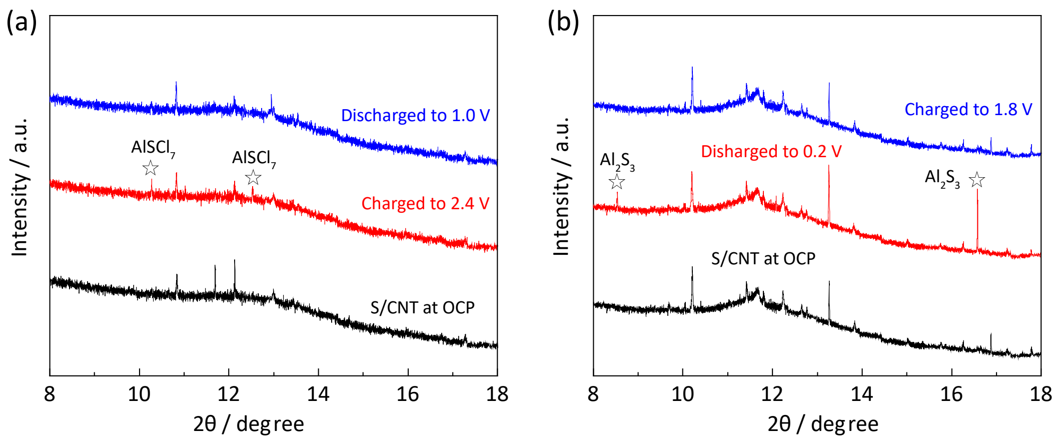

Supplementary Figure S5 The XRD patterns of S/CNT composite cathodes at different cut-off voltages based on (a) $AlSCl_7$ and (b) Al_2S_3 products. The corresponding wavelengths are 0.7290 nm and 0.6868 nm, respectively. For sulfur oxidation, the diffraction peaks of $AlSCl_7$ can be clearly observed at 2.4 V. These peaks disappear after discharging to 1.0 V due to the reversible conversion from $AlSCl_7$ to sulfur. For sulfur reduction, the peaks assigned to Al_2S_3 are seen at 0.2 V, and these peaks disappear at 1.8 V, suggesting the transformation from Al_2S_3 to sulfur during charge process.

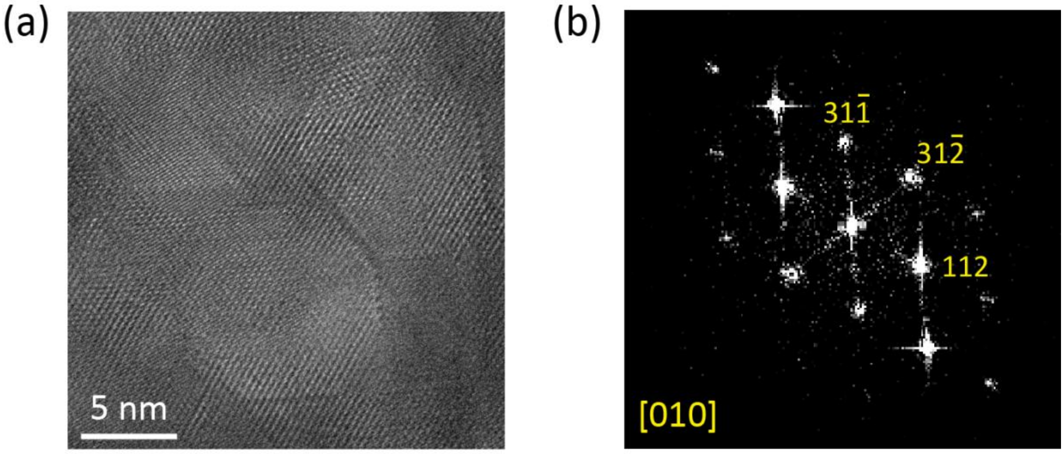

Supplementary Figure S6 (a) High-resolution TEM image of S_8; (b) The transformed FFT patterns.

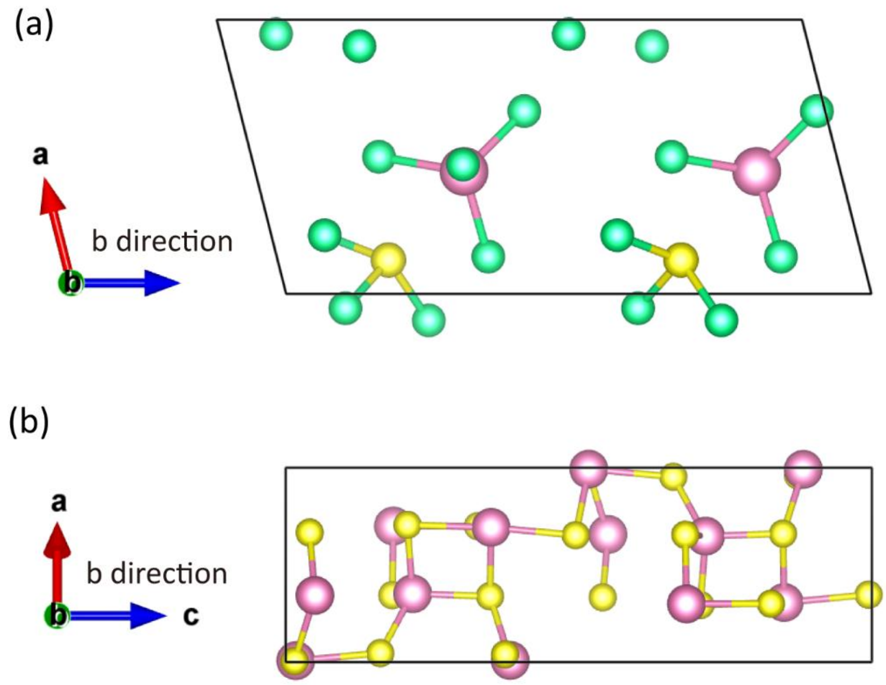

Supplementary Figure S7 The optimized models of $AlSCl_7$ and Al_2S_3. The angles between a-axis and c-axis correspond well with the STEM observation as shown in Figure 2b and 2d.

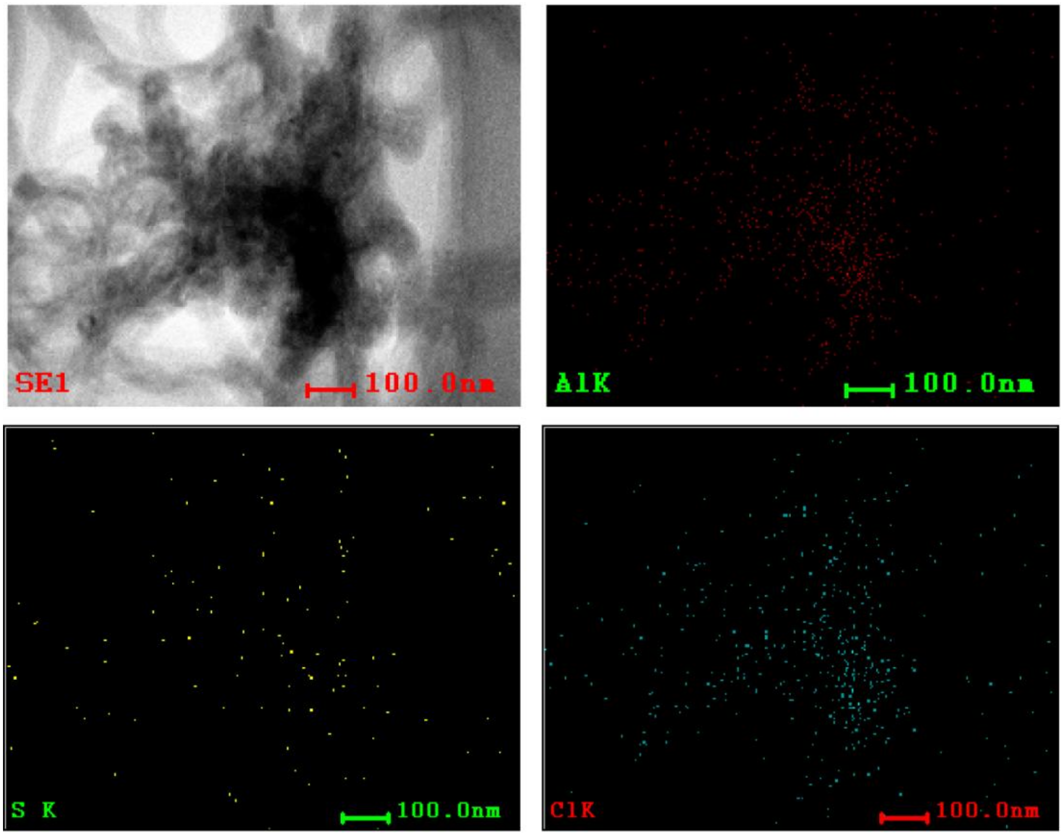

Supplementary Figure S8 TEM image of $AlSCl_7$ and its elemental mapping of Al, S, Cl. $AlSCl_7$ was obtained by disassembly of the Al−S battery after charging to 2.4 V. Obvious Al and Cl are observed on these images, suggesting the formation of $AlSCl_7$ during charging process.

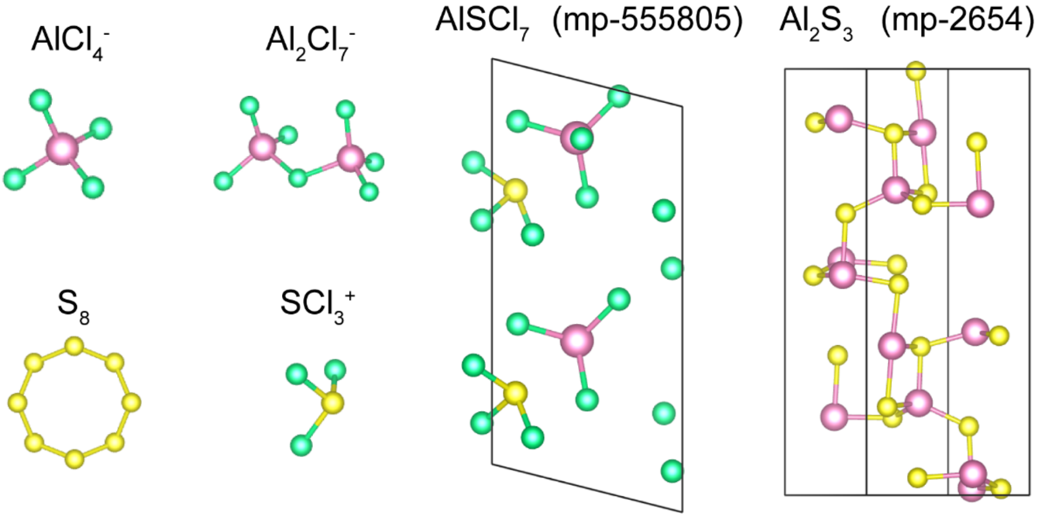

Supplementary Figure S9 The optimized theoretical structures of $AlCl_4^-$, $Al_2Cl_7^-$, S_8, SCl_3^+, $AlSCl_7$ and Al_2S_3.

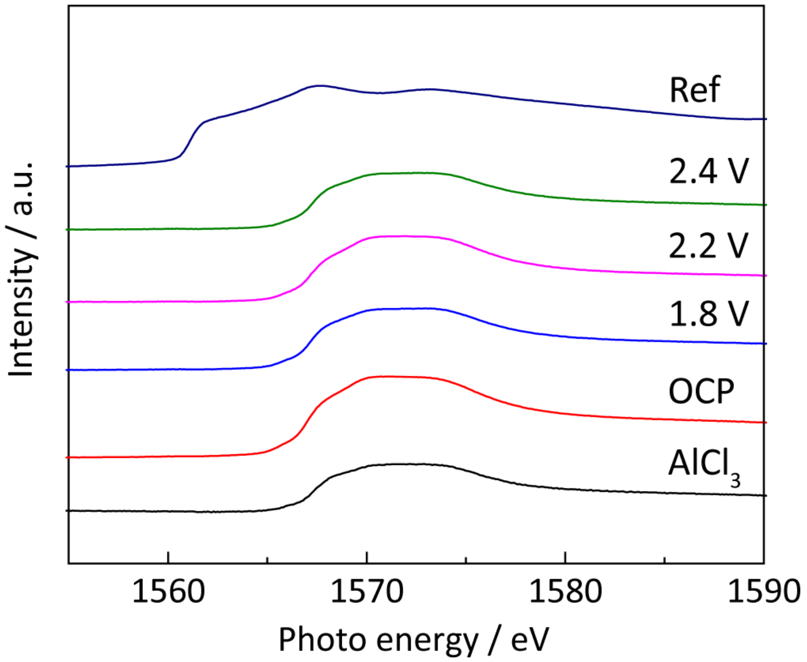

Supplementary Figure S10 The Al K edge NEXAFS spectra of sulfur cathodes at different charging potentials. Compared to the obvious peak shift of S and Cl (Figure 3a and 3b), no peak shift is visible during charging process. These results indicate that the Al valence remains unchanged during cycling.

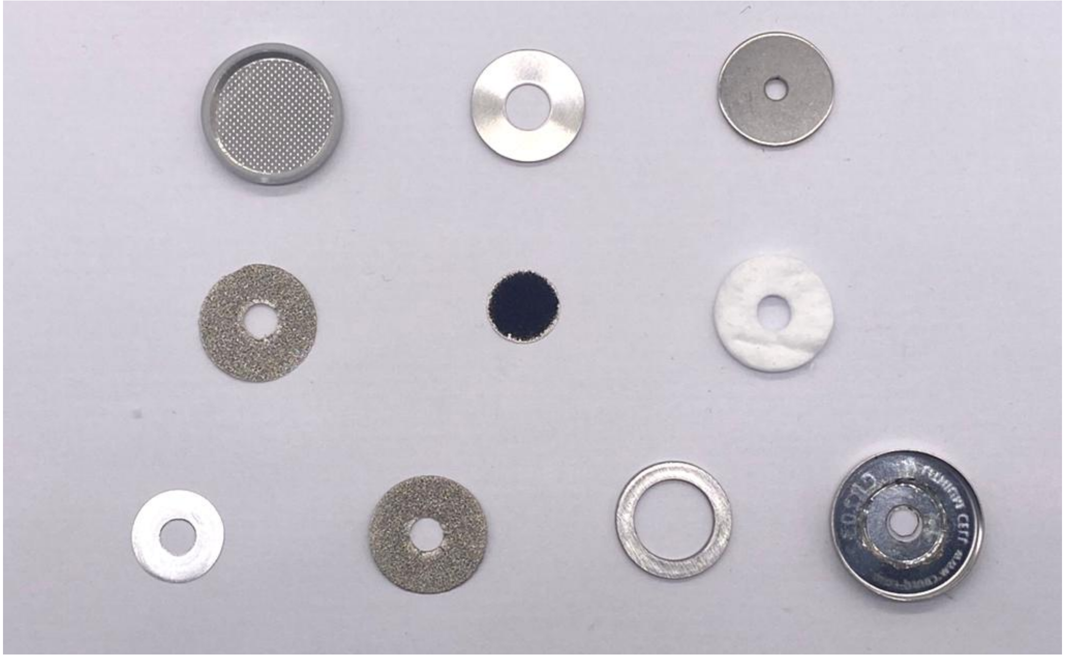

Supplementary Figure S11 The cell configurations for the *in-situ* Raman testing in Figure 4d.

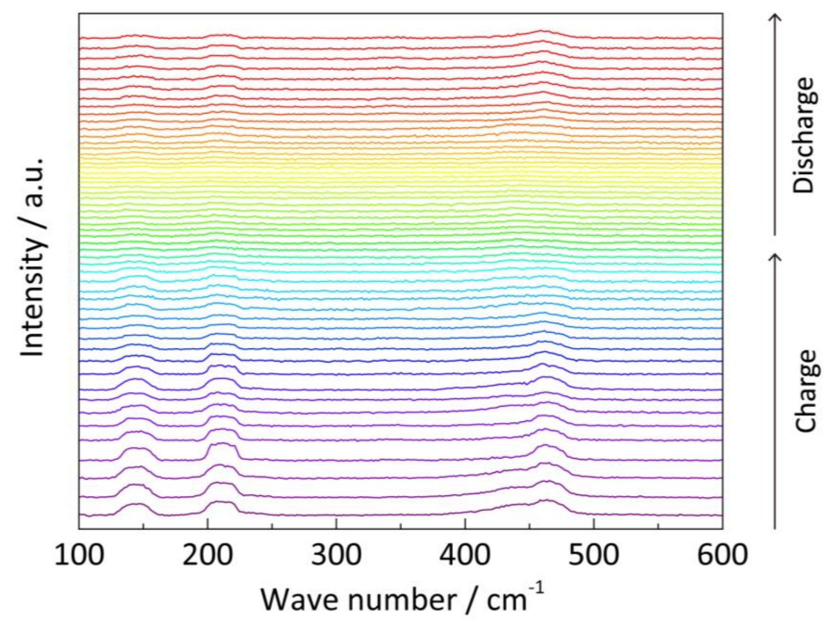

Supplementary Figure S12 The *in-situ* Raman spectra of sulfur cathode in Al−S battery. The decreased peak density of sulfur is visible during charging process, while the peaks emerge as the battery is discharged.

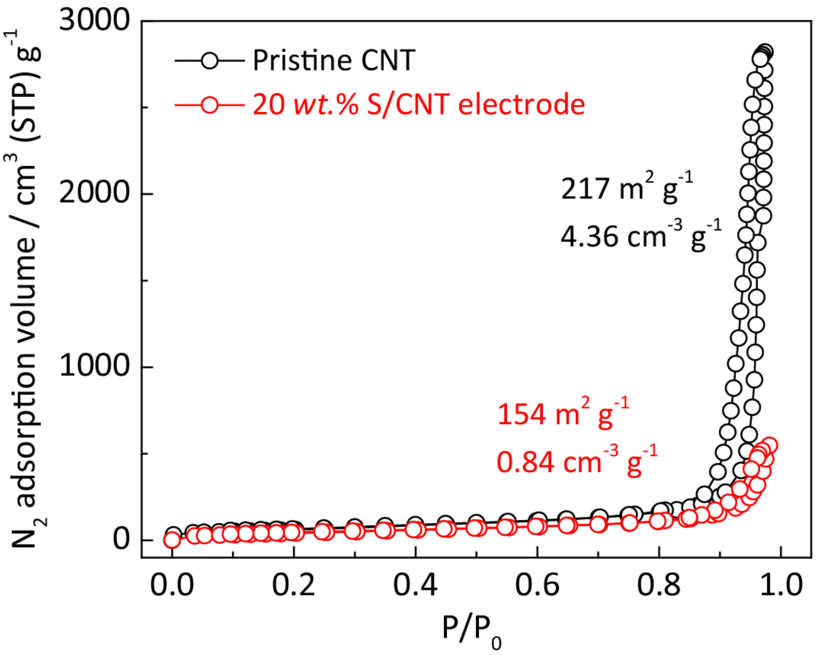

Supplementary Figure S13 N_2 adsorption-desorption isotherms (77 K) for pristine CNT and composite electrode with 20 *wt.*% sulfur in S/CNT. The pristine CNT shows a specific surface area of 217 m^2 g^{-1} with a pore volume of 4.36 cm^3 g^{-1}. After processing the S/CNT electrode with 20 *wt.*% sulfur, its specific surface area decreases to 154 m^2 g^{-1}, and meanwhile the pore volume decreases to 0.84 cm^3 g^{-1}. These decreases are attributed to the sulfur presence inside the CNT host.

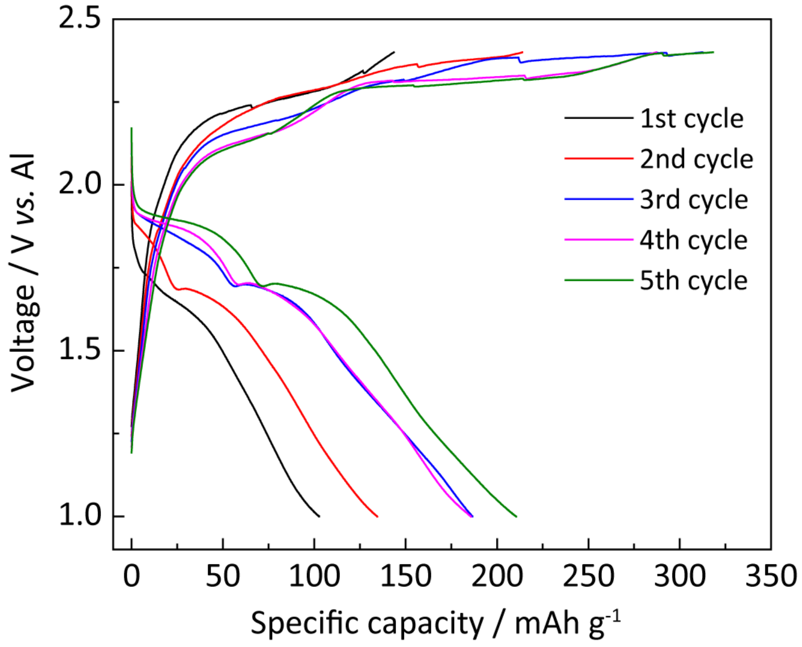

Supplementary Figure S14 The charge-discharge curves of Al−S batteries from 1st cycle to 5th cycle at 0.2 A g^{-1}. A pre-activation process with several electrochemical cycles is needed to activate the assembled fresh Al−S battery.

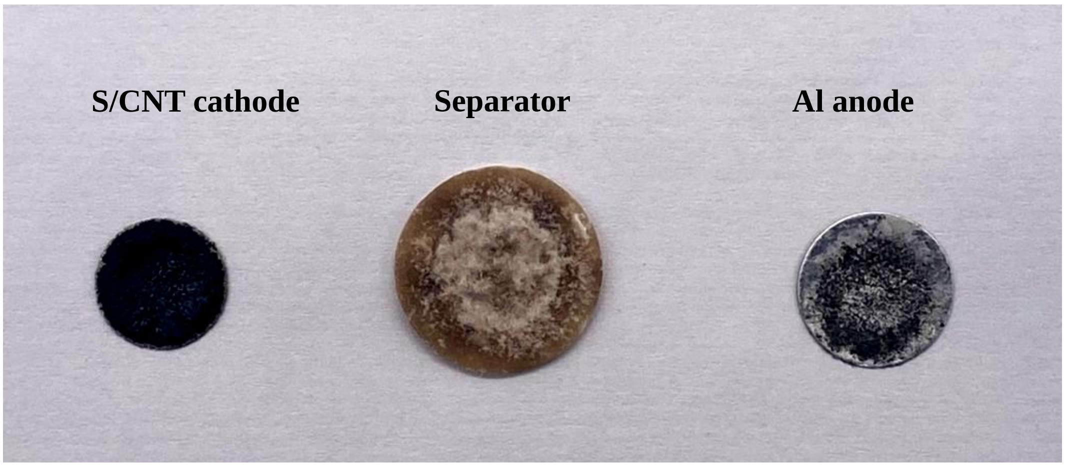

Supplementary Figure S15 Digital photos of sulfur cathode, separator and Al anode disassembled from the cycled Al−S batteries.

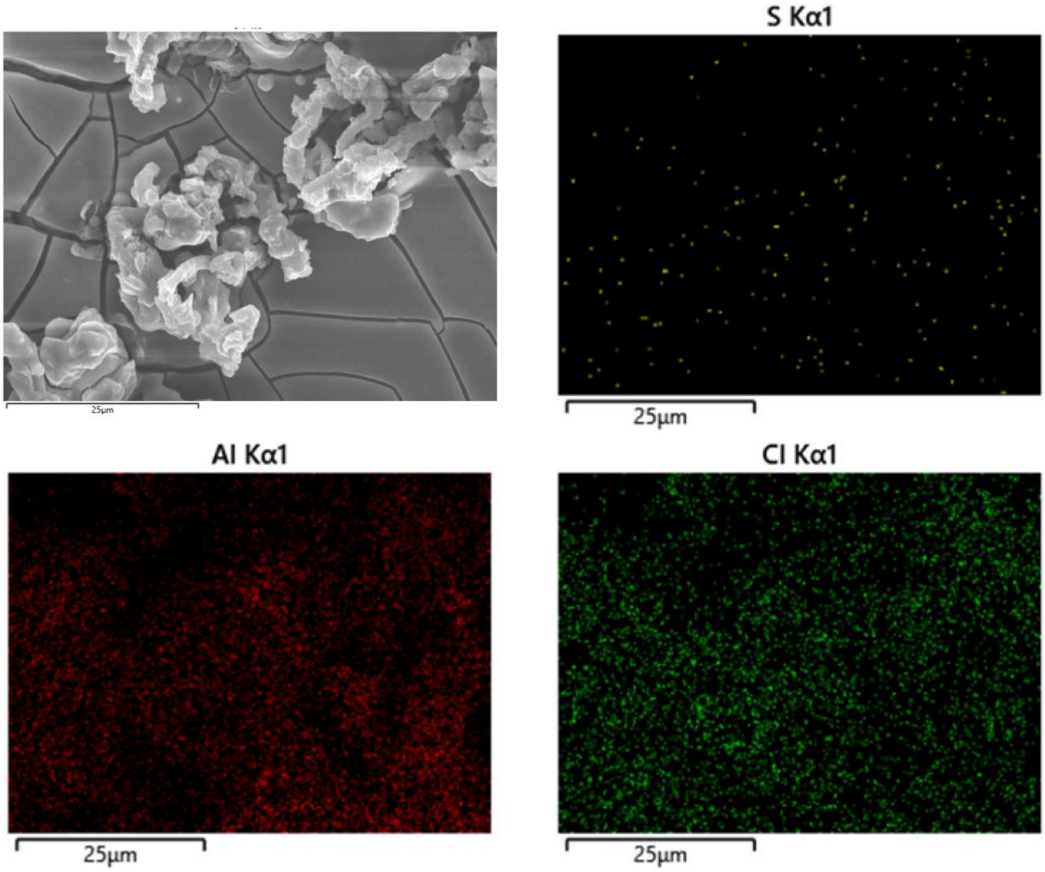

Supplementary Figure S16 The SEM image of cycled Al anode and its elemental mappings. There are obvious S and Cl signals for Al anode after cycling. This confirms the dissolution of SCl_3^+ and its migration to the anode side, leading to the capacity decay.

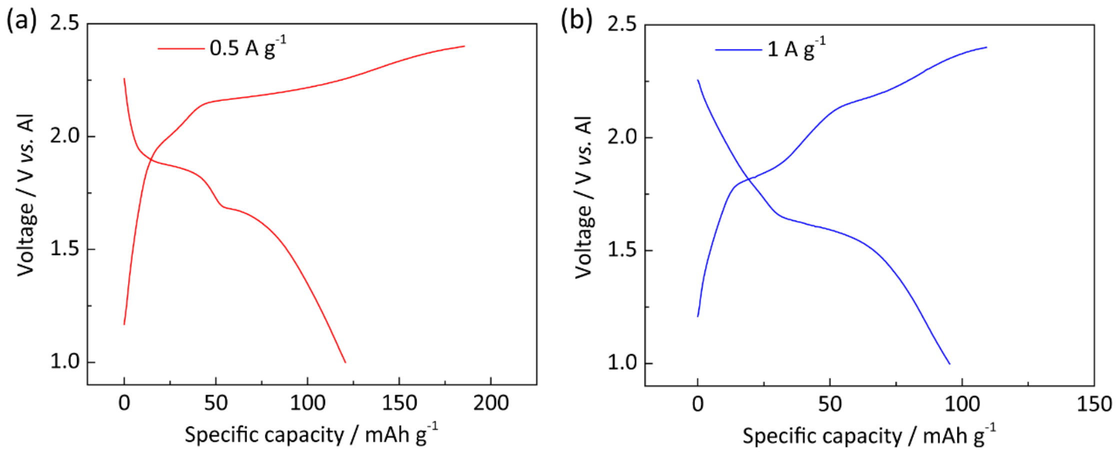

Supplementary Figure S17 Charge-discharge curves of Al−S batteries at 0.5 A g^{-1} and 1 A g^{-1}.

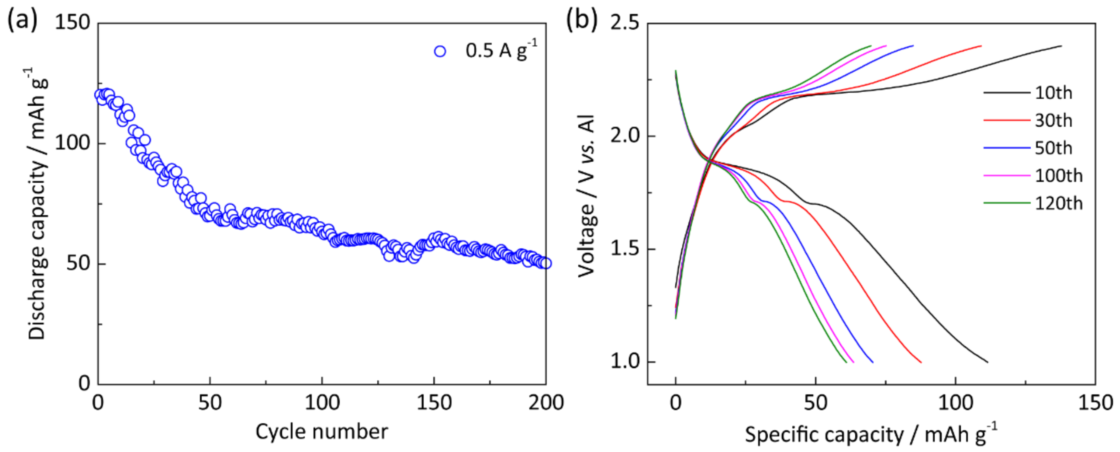

Supplementary Figure S18 (a) Cycling performance of Al−S battery at 0.5 A g^{-1}; (b) The charge-discharge curves at different cycles.

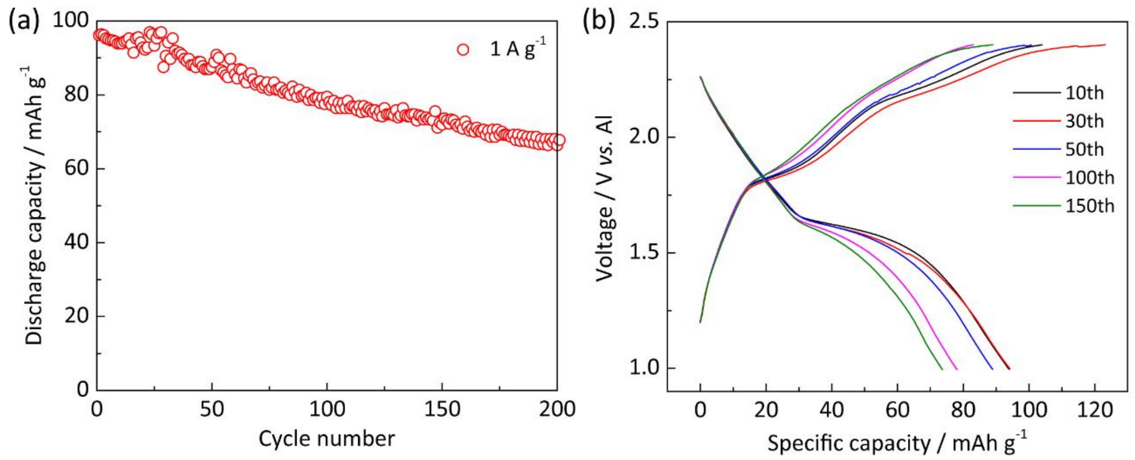

Supplementary Figure S19 (a) Cycling performance of Al−S battery at 1 A g^{-1}; (b) The charge-discharge curves at different cycles.

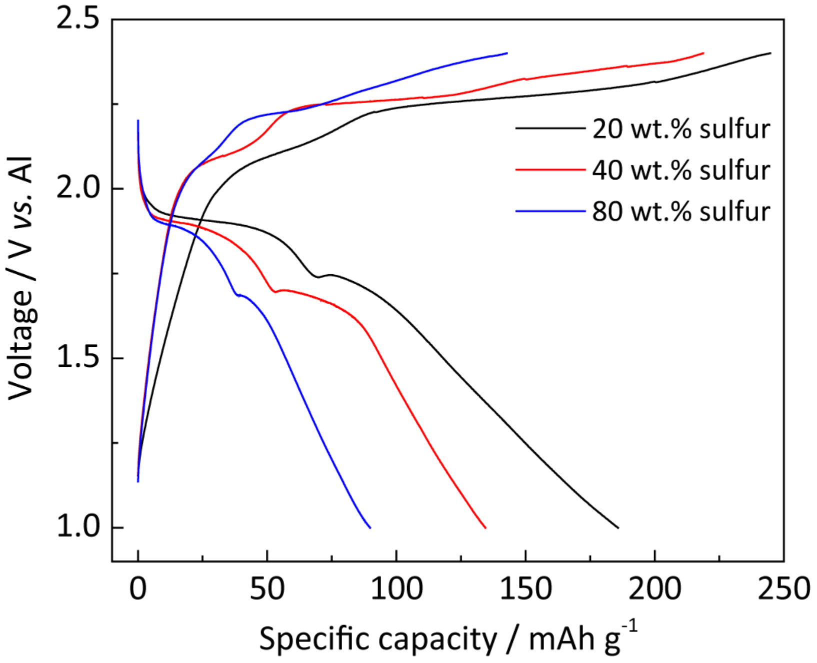

Supplementary Figure S20 Charge-discharge curves of Al−S batteries with different sulfur content in S/CNT composites. The applied current density is 0.2 A g^{-1}.

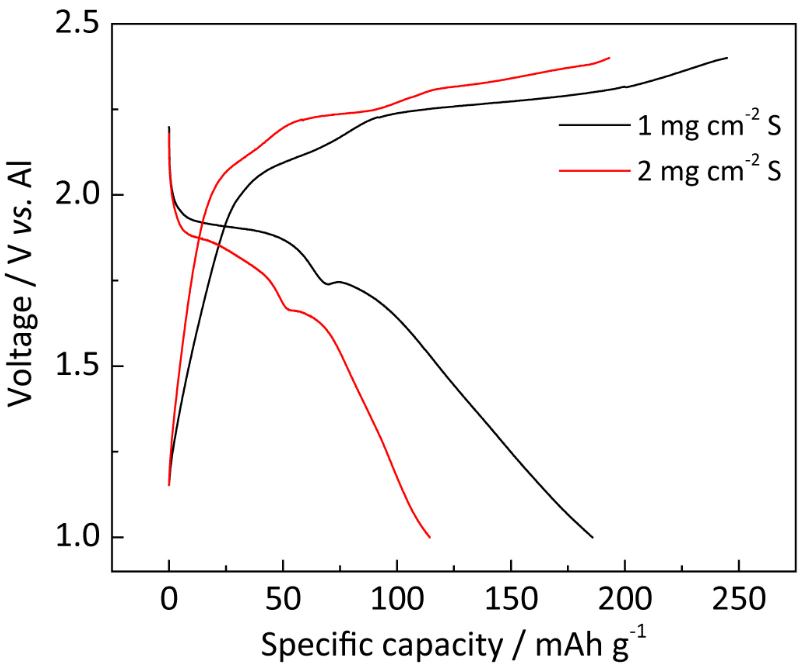

Supplementary Figure S21 Charge-discharge curves of Al−S batteries with different areal loadings of sulfur. The applied current density is 0.2 A g^{-1}.

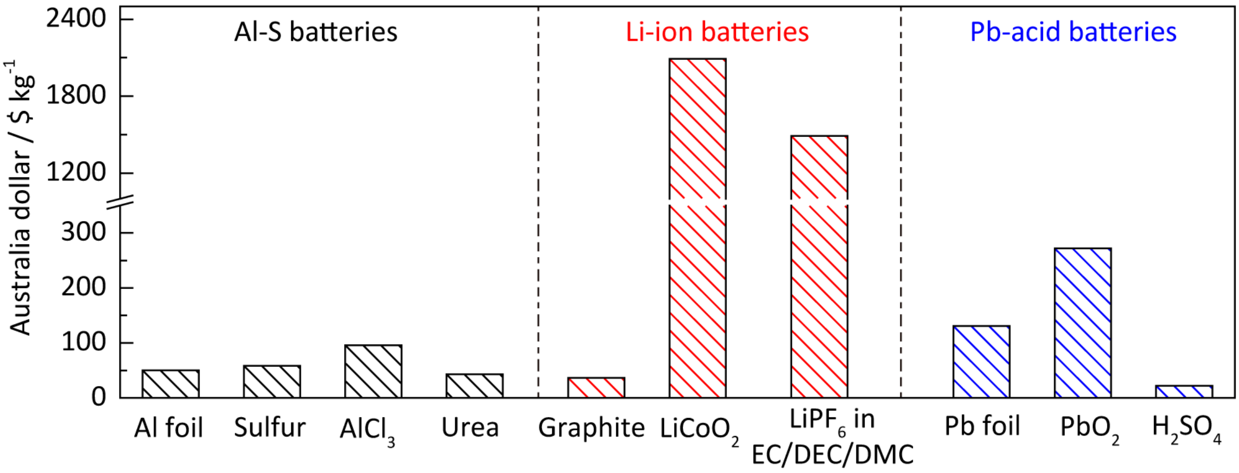

Supplementary Figure S22 The price comparison of electrode materials and electrolytes for Al−S, Li-ion and Pb-acid batteries.

Supplementary Table S1 The performance comparison with the reported cathode materials in ionic liquid electrolytes

Reported materials		Electrolyte	Discharge voltage / V	Specific capacity / mAh g^{-1}	Cycling life	Final capacity / mAh g^{-1}	Ref
Sulfur oxidation (This work)	S/CNT	$AlCl_3$/urea	~1.7 – 1.9 V	~225	200	~105	This work
Sulfur reduction	S/carbon cloth	$AlCl_3$/EmImCl	~ 0.6 V	~1300	20	~1000	1
	S/CNF paper	Li^+-$AlCl_3$/EmImCl	~ 0.75 V	~1000	50	~600	2
	S/CMK-3	$AlCl_3$/NBMPBr	~ 0.2 – 0.6 V	~1400	20	~600	3
	S/Co+C	$AlCl_3$/EmImCl	~ 0.1 – 0.8 V	~1600	200	~500	4
	S/CNF+coated separator	$AlCl_3$/EmImCl	~ 0.95 V	~1250	10	~600	5
Metal oxides	Binder-free V_2O_5	$AlCl_3$/BmImCl	~ 0.5 – 0.7 V	~240	5	~180	6
	V_2O_5 nanowires	$AlCl_3$/EmImCl	~ 0.55 V<br>~ 0.2 V	~305	20	~273	7
	Anatase TiO_2	$AlCl_3$/EmImCl	~ 0.5 V	~120	N/A	N/A	8
	Co_3O_4	$AlCl_3$/EmImCl	~ 0.2 – 0.7 V	~490	100	~122	9
Metal sulfides	Co_3S_4	$AlCl_3$/EmImCl	~ 0.7 V	~290	150	~90	10
	Mo_6S_8	$AlCl_3$/BmImCl	~ 0.55 V,<br>~0.38 V	~167	50	~70	11
	CuS	$AlCl_3$/EmImCl	~ 0.1 – 1.0 V	~250	100	~100	12
	MoS_2	$AlCl_3$/EmImCl	~ 0.4 – 0.8 V	~253	100	~67	13
Metal chlorides	VCl_3	$AlCl_3$/EmImCl	~ 1.0 V	~76	10	~10	14
Polymers	polypyrrole	$AlCl_3$/EmImCl	N/A	~70	100	~50	15
	polythiophene		~ 0.8 – 1.2 V	~90	100	~70	
Graphitic carbons	3D graphitic foam	$AlCl_3$/EmImCl	~ 1.5 – 2.2 V	~60	7500	~60	16
	Carbon paper	$AlCl_3$/EmImCl	~ 1.6 – 2.0 V	~85	50	~85	17
	Graphene nanoribbons	$AlCl_3$/EmImCl	~ 1.6 – 2.0 V	~110	10000	~123	18

Supplementary Table S2 The DFT-based energy (EDFT), zero-point energy (ZPE), entropy (TS) and Gibbs free energy (G) of different solids and ions

	Al	$AlCl_4^-$	$Al_2Cl_7^-$	$AlSCl_7$	Al_2S_3	S	Al^{3+}	SCl_3^+	Cl^-
E_{DFT}	-3.75	-22.43	-37.97	-28.1	-25.18	-4.13	-0.27	-3.54	-5.05
ZPE	0.032	0.12	0.27	0.315	0.25	0.041	0	0.04	0
TS	0.055	0.44	0.73	0.89	0.35	0.085	0.14	0.34	0.08
G	-3.77	-22.76	-38.43	-28.67	-25.28	-4.17	-0.42	-3.84	-5.13

Supplementary Table S3 The price list of electrode materials for Al−S, Li−ion and Pb−acid batteries based on the Sigma-Aldrich and Alfa Aesar catalogs.

**Battery**	**Components**	**Product**	**SKU-pack size**	**Price / AUS$ kg^{-1}**
**Al-S battery**	anode	Al foil	Z185140-1EA	50.2
	cathode	sulfur	13825-1KG-R	58.4
	electrolyte	$AlCl_3$	11019-6KG	96
		urea	U5128-5KG	43
**Li-ion battery**	anode	graphite	332461-12KG	36.4
	cathode	$LiCoO_2$	442704-100G-A	2090
	electrolyte	$LiPF_6$ in EC/DEC/DMC	901685-500ML	1490
**Pb-acid battery**	anode	Pb foil	GF03246465-2EA	131
	cathode	PbO_2	AC217530025 (from Alfa Aesar)	272
	electrolyte	H_2SO_4	1603131000	22

Supplementary References

1 Gao, T. *et al.* A rechargeable Al/S battery with an ionic-liquid electrolyte. *Angew. Chem. Int. Ed.* **55**, 9898-9901 (2016).

2 Yu, X., Boyer, M. J., Hwang, G. S. & Manthiram, A. room-temperature aluminum-sulfur batteries with a lithium-ion-mediated ionic liquid electrolyte. *Chem* **4**, 586-598 (2018).

3 Yang, H. *et al.* An aluminum-sulfur battery with a fast kinetic response. *Angew. Chem. Int. Ed.* **57**, 1898-1902 (2018).

4 Guo, Y. *et al.* Rechargeable aluminium-sulfur battery with improved electrochemical performance by cobalt-containing electrocatalyst. *Angew. Chem. Int. Ed.* **59**, 22963-22967 (2020).

5 Yu, X. & Manthiram, A. Electrochemical energy storage with a reversible nonaqueous room-temperature aluminum-sulfur chemistry. *Adv. Energy Mater.* **7**, 1700561 (2017).

6 Wang, H. *et al.* Binder-free V_2O_5 cathode for greener rechargeable aluminum battery. *ACS Appl. Mater. Interfaces* **7**, 80-84 (2015).

7 Jayaprakash, N., Das, S. K. & Archer, L. A. The rechargeable aluminum-ion battery. *Chem. Commun.* **47**, 12610-12612 (2011).

8 Koketsu, T. *et al.* Reversible magnesium and aluminium ions insertion in cation-deficient anatase TiO_2. *Nat. Mater.* **16**, 1142-1150 (2017).

9 Liu, J., Li, Z., Huo, X. & Li, J. Nanosphere-rod-like Co_3O_4 as high performance cathode material for aluminium ion batteries. *J. Power Sources* **422**, 49-56 (2019).

10 Li, H. *et al.* A highly reversible Co_3S_4 microsphere cathode material for aluminum-ion batteries. *Nano Energy* **56**, 100-108 (2019).

11 Geng, L., Lv, G., Xing, X. & Guo, J. Reversible electrochemical intercalation of aluminum in Mo_6S_8. *Chem. Mater.* **27**, 4926-4929 (2015).

12 Wang, S. *et al.* High-performance aluminum-ion battery with CuS@C microsphere composite cathode. *ACS Nano* **11**, 469-477 (2017).

13 Li, Z. Y., Niu, B. B., Liu, J., Li, J. L. & Kang, F. Y. Rechargeable aluminum-ion battery based on MoS_2 microsphere cathode. *ACS Appl. Mater. Interfaces* **10**, 9451-9459 (2018).

14 Suto, K. *et al.* Electrochemical properties of Al/vanadium chloride batteries with $AlCl_3$-1-ethyl-3-methylimidazolium chloride electrolyte. *J. Electrochem. Soc.* **163**, A742-A747

(2016).

15 Hudak, N. S. Chloroaluminate-doped conducting polymers as positive electrodes in rechargeable aluminum batteries. *J. Phy. Chem. C* **118**, 5203-5215 (2014).

16 Lin, M.-C. *et al.* An ultrafast rechargeable aluminium-ion battery. *Nature* **520**, 324-328 (2015).

17 Sun, H. B. *et al.* A new aluminium-ion battery with high voltage, high safety and low cost. *Chem. Commun.* **51**, 11892-11895 (2015).

18 Yu, X., Wang, B., Gong, D., Xu, Z. & Lu, B. Graphene nanoribbons on highly porous 3D graphene for high-capacity and ultrastable Al-ion batteries. *Adv. Mater.* **29**, 1604118 (2017).
